# Large-scale mapping of bioactive peptides in structural and sequence space

**DOI:** 10.1371/journal.pone.0191063

**Published:** 2018-01-19

**Authors:** Agustina E. Nardo, M. Cristina Añón, Gustavo Parisi

**Affiliations:** 1 Departamento de Ciencia y Tecnología, Universidad Nacional de Quilmes, CONICET, Bernal, Argentina; 2 Centro de Investigación y Desarrollo en Criotecnología de Alimentos, Facultad de Ciencia Exactas, Universidad Nacional de la Plata - Comisión de Investigaciones Científicas - CONICET, La Plata, Argentina; Swiss Institute of Bioinformatics, SWITZERLAND

## Abstract

Health-enhancing potential bioactive peptide (BP) has driven an interest in food proteins as well as in the development of predictive methods. Research in this area has been especially active to use them as components in functional foods. Apparently, BPs do not have a given biological function in the containing proteins and they do not evolve under independent evolutionary constraints. In this work we performed a large-scale mapping of BPs in sequence and structural space. Using well curated BP deposited in BIOPEP database, we searched for exact matches in non-redundant sequences databases. Proteins containing BPs, were used in fold-recognition methods to predict the corresponding folds and BPs occurrences were mapped. We found that fold distribution of BP occurrences possibly reflects sequence relative abundance in databases. However, we also found that proteins with 5 or more than 5 BP in their sequences correspond to well populated protein folds, called superfolds. Also, we found that in well populated superfamilies, BPs tend to adopt similar locations in the protein fold, suggesting the existence of hotspots. We think that our results could contribute to the development of new bioinformatics pipeline to improve BP detection.

## Introduction

As several endocrine and nervous systems in mammals are regulated by endogenous peptides, many of them could also be regulated by exogenous peptides performing hormone-like functions [1]. Due to their physiological importance and regarding their nutritional values, these peptides are called bioactive peptides or just biopeptides (BP) [2]. In the last years, several works from food science and biotechnological areas have shown that several food proteins contain different amounts of these exogenous peptides [3,4]. In general, these peptides are between 3 and 20 amino acids long and are found as encrypted regions in protein sequences. During the gastrointestinal digestion, these peptides are released from proteins by the action of digestive enzymes and could be absorbed through the intestine to enter the blood circulation. The pathway and the absorption mechanism differ according the length and charge of the peptide. In intestinal light di and tripeptides are uptake into the enterocyte through the intestinal endothelium by the co-transporter PepT1[5]. There is also evidence that some di and tripeptides can survive cytosolic hydrolysis and be transported intact through the basolateral membrane [6]. Other peptides can (although in a much smaller proportion) diffuse through tight junctions and / or enter through vesiculation [7]. Releasing process could also be reproduced *in vitro* conditions which allow their purification and biochemical characterization [8,9]. Once in blood circulation, BP can modulate the biological activity of several human enzymes playing key roles in different metabolisms such as the regulation of blood pressure, stimulating or suppressing the action of the immune system, modulating the activity of the nervous system, showing an anti-inflammatory effect and inducing a reduction in cholesterolaemia among others [10]. It has been found that biological effects of BP mostly reside on their sequence or primary information, but could also be related with composition [11,12] and with their spatial arrangements [13,14]. In general they exert just one biological activity but it has been described that some could have multifunctional activities [15].

Due to their health-enhancing potential, BP research has been especially active to use them as components in functional foods or nutraceuticals [3,16,17]. A functional food is a natural o processed food which contains a biologically-active compounds, which in defined amount benefits a limited number of functions in the body providing welfare and health benefit for the prevention, management, or treatment of chronic disease [18]. In this sense, several databases and bioinformatics tools have been developed to predict and study BP occurrence in food proteins. Among the databases, we found PepBank [19], Antimicrobial Peptide Database (APD) [20] and BIOPEP [21]. Databases are the main source of information to predict occurrence of BP in proteins. Basically, primary structure information of characterized peptides is used to search for sequence similarity to predict occurrence of BP in different proteins. Sequence similarity searches are commonly performed using BLAST and PSI-BLAST programs [22]. Also, primary structure could be also used to train machine learning algorithms to predict occurrence of BP [23] and also for simulation of BP release by the action of digestive enzymes in a digestive in silico simulation process [24]. Sequence similarity searches between BP and target sequences then are the major methodologies to estimate the presence of BP. This is inherently connected with the nature of BP in the containing proteins. Apparently, the BP encoded in food proteins does not have a given biological function in those proteins and for that reason do not evolve under given evolutionary constraints (i.e. to preserve structure, function, stability). This fact reveals the main difficulties observe to predict occurrence of BP in proteins since most bioinformatics tools take advantage of differential patterns of amino acid substitution (i.e. conservation or coevolution) to discover and predict special important regions in proteins.

In this work we perform a large-scale characterization of BP in sequence and structural space. We search for exact occurrences of BP in non-redundant database. We retrieved all the protein sequences containing at least one occurrence of BP which was then submitted to a fold assignment pipeline using CATH database [25]. With those sequences with a detected structural template, we mapped BP positions on the structure representing all the members of each homologous superfamily detected in this study. Two main results emerged from our study. We found that some superfamilies show co-localization of several BP in their structures showing same or different biological activity. Also we found that proteins with more than 5 BPs in their sequences belongs to superfamilies with great structural and sequence variability as derived from CATH superfamilies analysis. Both results could help in the design of bioinformatic tools to localize given BPs and given structural superfamilies and also to select proteins having larger probabilities to contain BP in food protein screening.

## Materials and methods

Using BIOPEP database (http://www.uwm.edu.pl/biochemia/index.php/pl/biopep) [21] of bioactive peptides, we have selected each peptide above 5 residues long to perform a BLAST sequence search in nr database. From these results, we selected those sequence having exact matches for all the residues in each peptide. To reduce redundancy of the obtained sequences we used CD-Hit program (http://weizhongli-lab.org/cd-hit/) [26] at 100% cut-off to eliminate all duplicated sequences. We then performed a fold assignment for each of the sequence. For this purpose, we used BLAST searches (BLASTP) using default parameters against CATH database (http://www.cathdb.info/) [27]. For each sequence we selected best CATH domains matches (each with a E-value below 1x10^-3^) corresponding to non-overlapping segments of each sequence to assign a CATH domain in the case the protein have more than two structural domains. We also searched among the results for those segments having a peptide to assign a CATH domain. For each CATH domain identified in our dataset, we have used the information derived for the Funtree database [28] as well as CATH specific annotation (http://www.cathdb.info/) to studied GO terms annotations.

**Fig 1:**
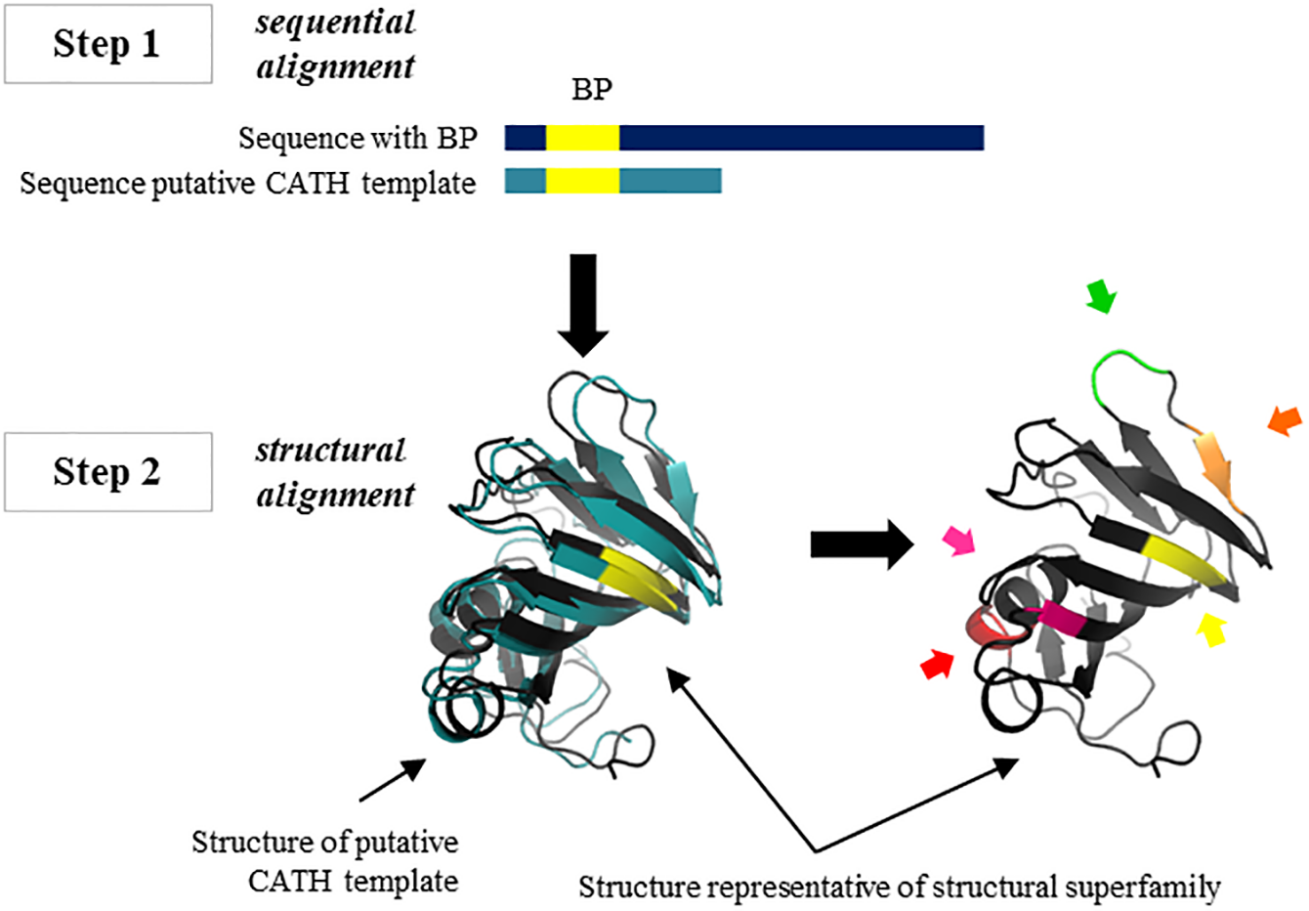
Diagram of structural mapping of the bioactive peptides on structural superfamilies. Once a BP was detected in a given protein after sequence exact searches, we identified putative folds for that sequence using fold-assignment techniques. Templates in this fold identification were those domains deposited in CATH database. We then structurally aligned the sequence putative CATH template with the corresponding representative protein of the structural superfamily again accordingly with CATH database. We finally mapped the BP occurrence to the representative template of each structural superfamily.

For those sequences where was possible to assign a given fold, we then proceed to perform a structural mapping of the peptides on putative templates. For this purpose, we performed a two steps procedure (Fig 1). First, we aligned each sequence with its corresponding putative template, indicated by the CATH domain ID, using ClustalX [29]. We then structurally aligned the CATH domain (template) with the protein fold which is taken as representative of corresponding structural superfamily, following CATH, using the program ProFit (http://www.bioinf.org.uk/programs/profit/index.html). In CATH database structural superfamilies have the same first four CATH number (Class, Architecture, Topology and Homologous numbers characterize a given structural superfamily). Each superfamily could have several homologous families characterized by the fifth; sixth and so on CATH id numbers indicating levels of sequence conservation (for example, S35 represent the number of homologous families sharing more than 35% identity between the corresponding members). With this procedure, we were able to map peptides in a unique protein fold representing different proteins for the same homologous family. Each protein fold with all the mapped peptides were identify with the four first numbers corresponding to CATH database. In addition, we used CATH derived information to characterize the structural superfamily divergence using the number of structural clusters and the number of S35 homologous families in each structural superfamily.

## Results and discussion

### Mapping biopeptides in sequence and structural space

**Fig 2:**
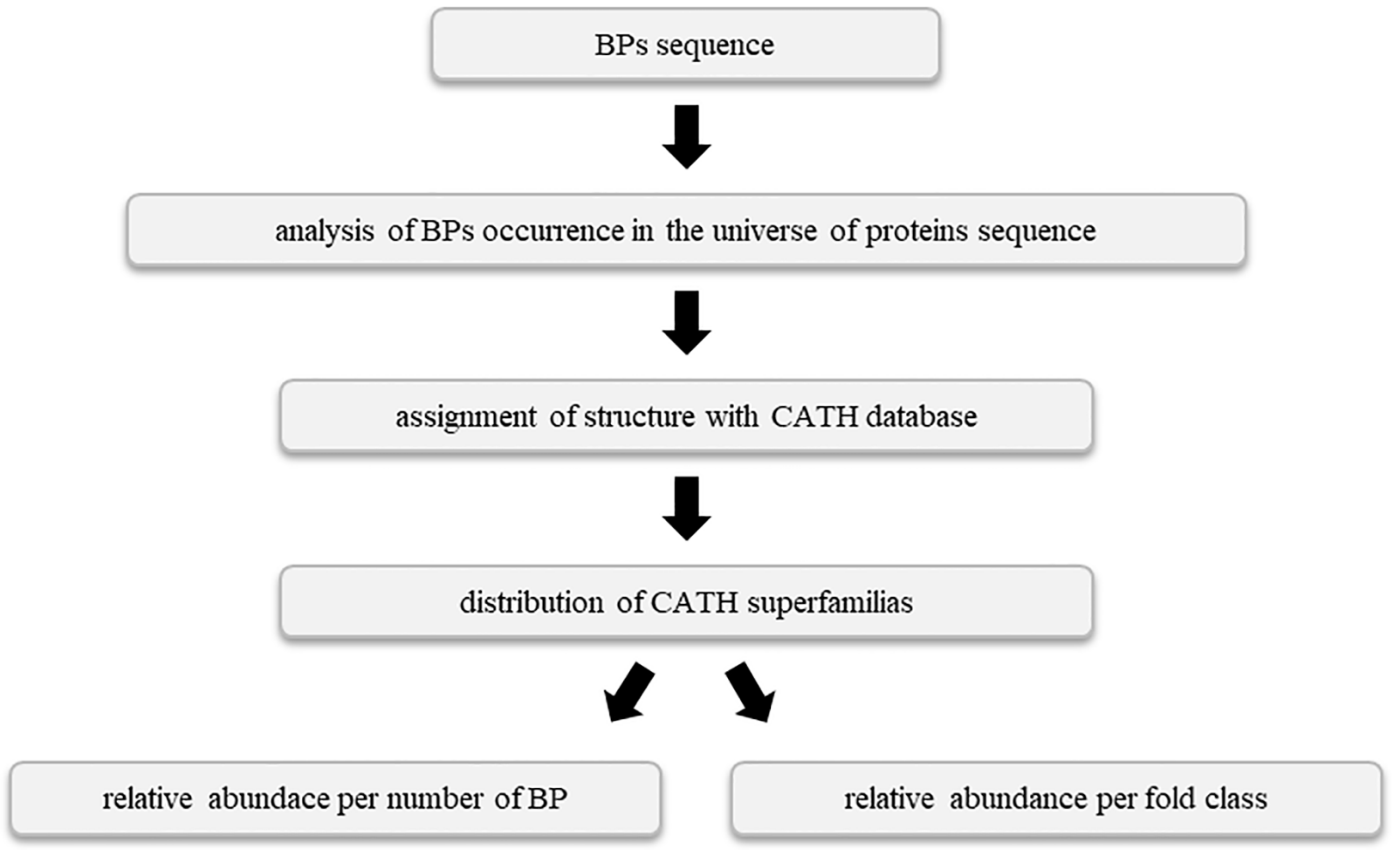
Flowchart of main procedure followed to mapping the structural occurrence of biopeptides.

To assess how structural information could be used in the identification of bioactive peptides, we performed a large-scale database sequence search followed by a structural mapping. Using BLAST searches against non-redundant database, we searched for proteins containing exact matches for each peptide more than 5 residues long taken from BIOPEP database [21]. Using 2595 peptides as input for similarity searches, we retrieved 88909 protein sequences with at least one occurrence of a given BP. We used the program CD-HIT [26] to reduce the redundancy of the set removing 100% identity sequences, remaining 80523 unique sequences. We then performed a fold assignment for each of these sequences using BLAST searches against sequences corresponding to structural domains contained in CATH database. CATH database is a structural database of all known protein structures chopped as structural domains. CATH classified each domain with a series of numbers to identify its structural class (C), architecture (A), topology (T) and homologous superfamily (H). As proteins in our dataset could contain multiple structural domains, we only assigned a putative template for those containing peptides. From BLAST searches against CATH domains, we selected those containing E-values less than 1x10^-3^. Using this method, we were able to assign a putative template to 58167 sequences (72.2%). Sequences and putative templates were then aligned using ClustalX [29]. In order to map the occurrence of peptides in the corresponding protein fold, we structurally aligned templates with the representative protein fold corresponding to the homologous superfamily taken from CATH. Using this multiple alignment between a sequence containing the exact match of a given bioactive peptide, its corresponding putative template, and the representative protein fold for the corresponding structural family; we proceeded to map the location of the bioactive peptide on the representative fold. This procedure was followed for each protein in our dataset. A flowchart of all these procedure is shown in Fig 2.

**Fig 3:**
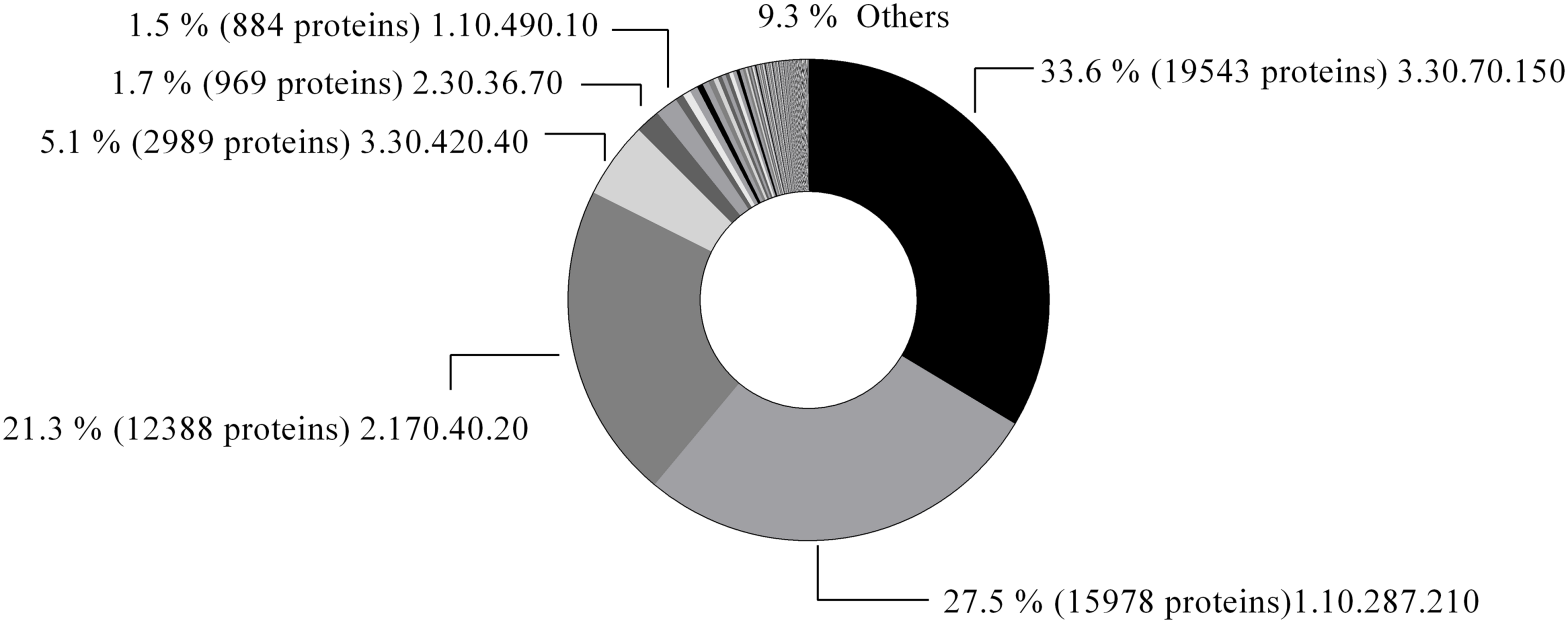
Distribution of protein folds that contains biopeptides extracted from BIOPEP database.

Using CATH domain classification, we found that all sequences clustered in 333 domain superfamilies. In Fig 3 and Table 1 we show the obtained distribution of proteins folds.

**Table 1:** Description of the top ten most abundant superfamilies found in the assignation of structure of the sequences with at least one peptide.

Percentage of proteins with assigned fold	CATH	Representative domain in CATH	C	A	T	Representative protein
**33.60**	3.30.70.150	1wddA01	Alpha Beta	2-Layer sandwich	Alpha-Beta plaits	Large subunit rubisco
**27.47**	1.10.287.210	1qbzA00	Mainly Alpha	Orthogonal bundle	Helix hairpins	Envelope glycoprotein gp160
**21.30**	2.170.40.20	1kmoA02	Mainly Beta	Beta barrel	Maltoporin; chain A	HIV envelope protein Gp120; chain G
**5.14**	3.30.420.40	3i33A01	Alpha Beta	2-Layer Sandwich	Nucleotidyl transferase; domain 5	Plasmodium falciparum actin I
**1.52**	1.10.490.10	2nrlA00	Mainly Alpha	Orthogonal Bundle	Globin-like	Blackfin tuna myoglobin
**1.67**	2.30.36.70	1s22A02	Mainly Beta	Roll	Actin; chain A, domain 2	Actin; chain A, domain 3
**0.55**	3.40.190.10	1ixhA01	Alpha Beta	3-Layer(aba) Sandwich	D-maltodextrin binding protein; domain 2	Periplasmic binding protein-like II
**0.54**	2.60.40.720	2ioiA00	Mainly Beta	Sandwich	Immunoglobulin-like	Human mutant, p53
**0.53**	3.40.50.720	1c0pA01	Alpha Beta	3-Layer(aba) Sandwich	Rossmann fold	NAD(P)-binding Rossmann-like domain
**0.42**	2.60.120.10	1juhA02	Mainly Beta	Sandwich	Jelly rolls	Jelly rolls

It is interesting to note that four main folds accounts for the 87.54% of the sequences. The most populated cluster with 33.6% of the sequences corresponds to the fold with CATH id 3.30.70.150, an alpha-beta 2-layers sandwich. This fold is represented by ribulose bisphosphate carboxylase large chain (RuBisCO large subunit) (EC 4.1.1.39) and most proteins are close homologous to RuBisCO. A minor percentage of the proteins are represented by a relative of RuBisCO, the 2,3-diketo-5-methylthiopentyl-1-phosphate enolase (DK-MTP-1-P enolase) (EC 5.3.2.5) the so-called RuBisCO-like protein. Almost 96% of the proteins in this cluster belong to plants and the rest to Bacteria, mainly Cyanobacterias. This cluster is followed in abundance by a mainly alpha, orthogonal bundle (CATH id 1.10.287.210) which accounts for the 27.5% of the sequences. The 92.8% of these sequences in this cluster belongs to Viruses and are represented by the protein Envelope glycoprotein gp160 (Envpolyprotein). A mainly beta fold with a complex beta structure (CATH id 2.170.40.20) accounts for the 21.3% of sequences. These proteins belong to the so-called Envelope glycoprotein gp160 (Envpolyprotein). Finally, the family representing the Actin (beta-actin) and actin-related proteins (CATH id 3.30.420.40) accounts for the 5.14% of the sequences in the dataset. In Table 1 we show the top 10 protein superfamilies structurally clustering the sequences used in this study. It is evident that these distributions do not reflect any particular property of folds containing BP (differential stability, special structure-function relationship, etc.), but just the sequence distribution in the database derived from genome sequence projects. From these most populated folds, the Actin fold (CATH id 3.30.420.40) is the only belonging to the class denominated “superfold” [30], which are those folds associated to a large number of families and functions. We will see below the importance of superfolds in BP biology.

### Mapped biopeptides and structural superfamilies

The distribution of mapped biopeptides on protein folds changes when we considered the number of different biopeptides associated to each structural domain. The structural families CATH id 3.30.70.150, 3.30.420.40 and 1.10.287.210 mentioned above, are only associated with just one biopeptide but the family 3.30.420.40 with 7 different biopeptides.

When we studied the occurrence of different biopeptides in a given structural domain, we found that a given protein fold could contain several biopeptides with the same or different associated functions. In S2 Table we show the number of biopeptides associated with a given protein fold, showing only the top containing proteins. It is then possible to observe that the fold with CATH ID 3.40.50.300 (P-loop containing nucleotide triphosphate hydrolases superfamily) contains 35 different biopeptides showing 12 different biological activities being the maximum number of different peptides mapped in a given fold. This distribution falls to 197 different folds just containing the occurrence of only 1 BP.

**Fig 4:**
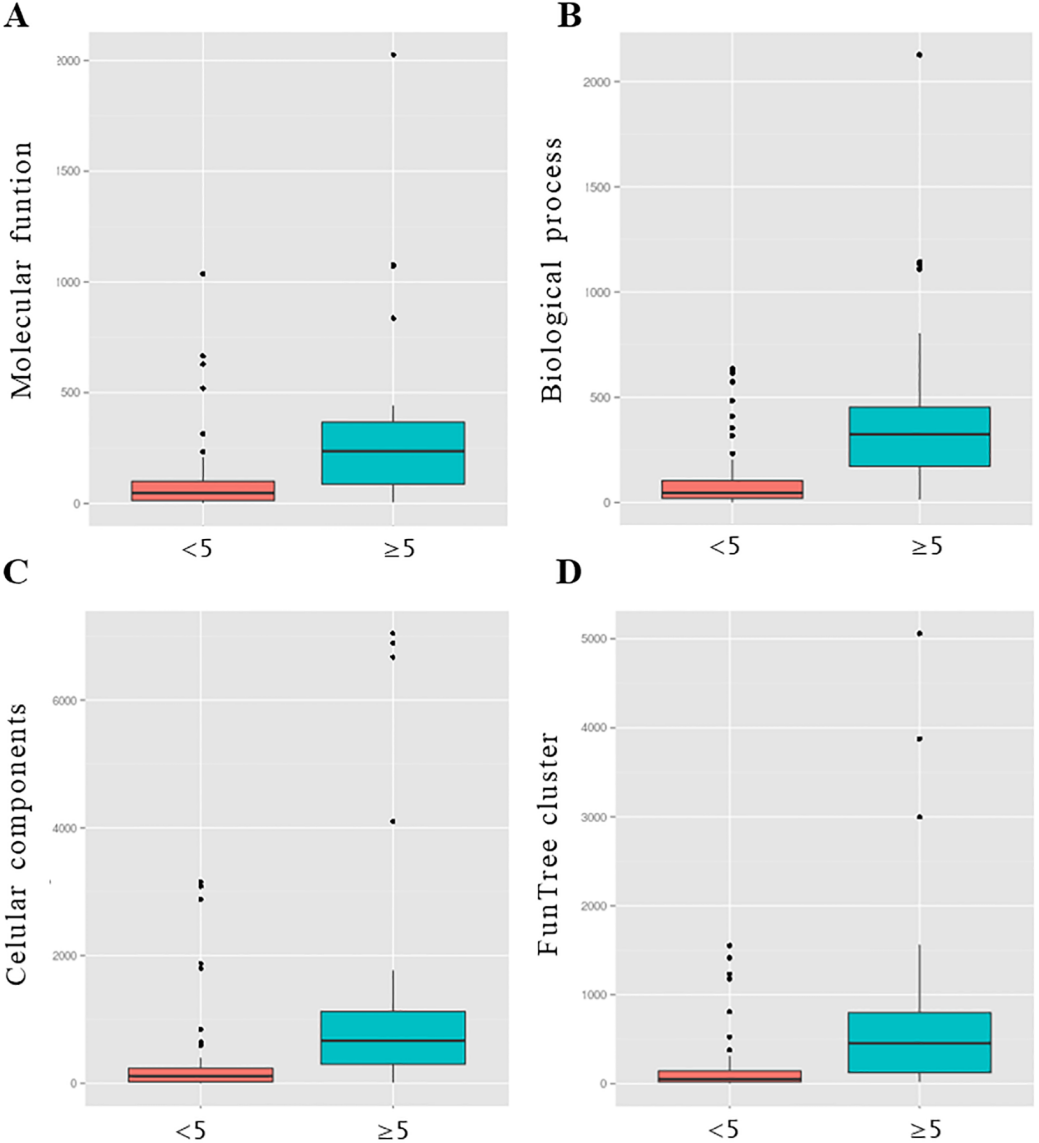
Distribution of number of GO terms associated with folds with more and less than 5 biopeptides. Distributions of different GO terms per each class (Molecular function, Cellular components and Biological process) for proteins showing less than 5 and 5 or more BP (panels a, b, and c). Panel d, shows the same distribution but now using FunTree clusters information. In all the cases it is possible to observe that proteins with more than 5 BPs are functionally more diverse than those proteins with less than 5 BPs.

When we studied GO terms annotations [31] of each CATH domain as a function of the number of mapped biopeptides per structural domain, we found that proteins associated with larger number of biopeptides are functionally more diverse. In Fig 4 we show the distribution of GO terms associated with folds with more and less than 5 biopeptides. Using the three types of GO terms (molecular function, biological process and cellular components) folds with more than 5 biopeptides appear functionally more diverse than those sequences with fewer occurrences of BP (Fig 4a, 4b and 4c). We obtained a similar result using the functional clusters derived in Funtree [28] which is also associated with CATH database. In Fig 3d, we show the distribution for the number of different functional clusters which again show more functional diversification for those proteins associated with more than 5 biopeptides. Interestingly, the distribution of sequences lengths for the proteins with more and less than 5 BP is statistically different (Two-sample Kolmogorov-Smirnov test p-value < 0.001) but unexpectedly proteins with more BP are shorter.

**Fig 5:**
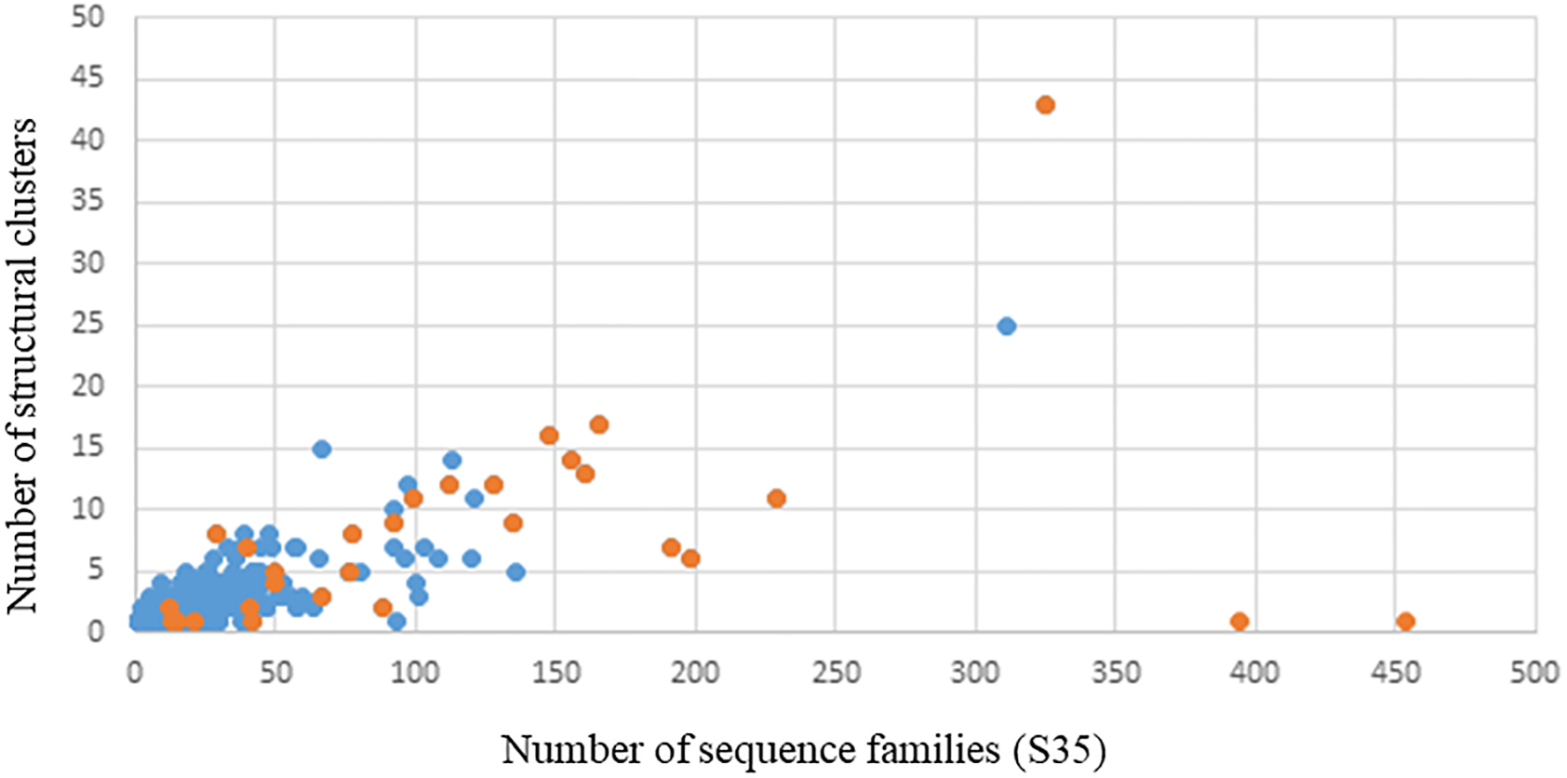
Number of different structural and sequential clusters derived from CATH database. Blue dots represent proteins with less than 5 BPs and the orange ones those with 5 or more BPs. It is possible to see that proteins with more than 5 BPs are more diverse structurally and sequentially.

When we studied the relationship of sequence divergence to structural conservation we found that proteins associated to more than 5 biopeptides per fold have higher number of sequences per protein fold but also a larger number of structural clusters (Fig 5). Both measures are indicative of higher evolutionary divergence in their evolutionary process. This relationship was studied using the number of protein families sharing more than 35% identity (S35 number in CATH) and the number of structural cluster in the corresponding structural superfamily. It is interesting to note, that only two protein families corresponding with 5 or more biopeptides mapped have just only one structural cluster (families 3.40.50.720 and 2.60.40.10 see Fig 5) but with a large number of associated S35. These families correspond to a Rossman-like and the Inmunoglobulin domains respectively. As we mentioned above, these domains are called “supefolds” [30] because they are very well conserved from the structural point of view but are extremely diverse in the sequence and functional dimension.

### Mapped biopeptides and putative hot-spots of protein structural superfamilies

**Fig 6:**
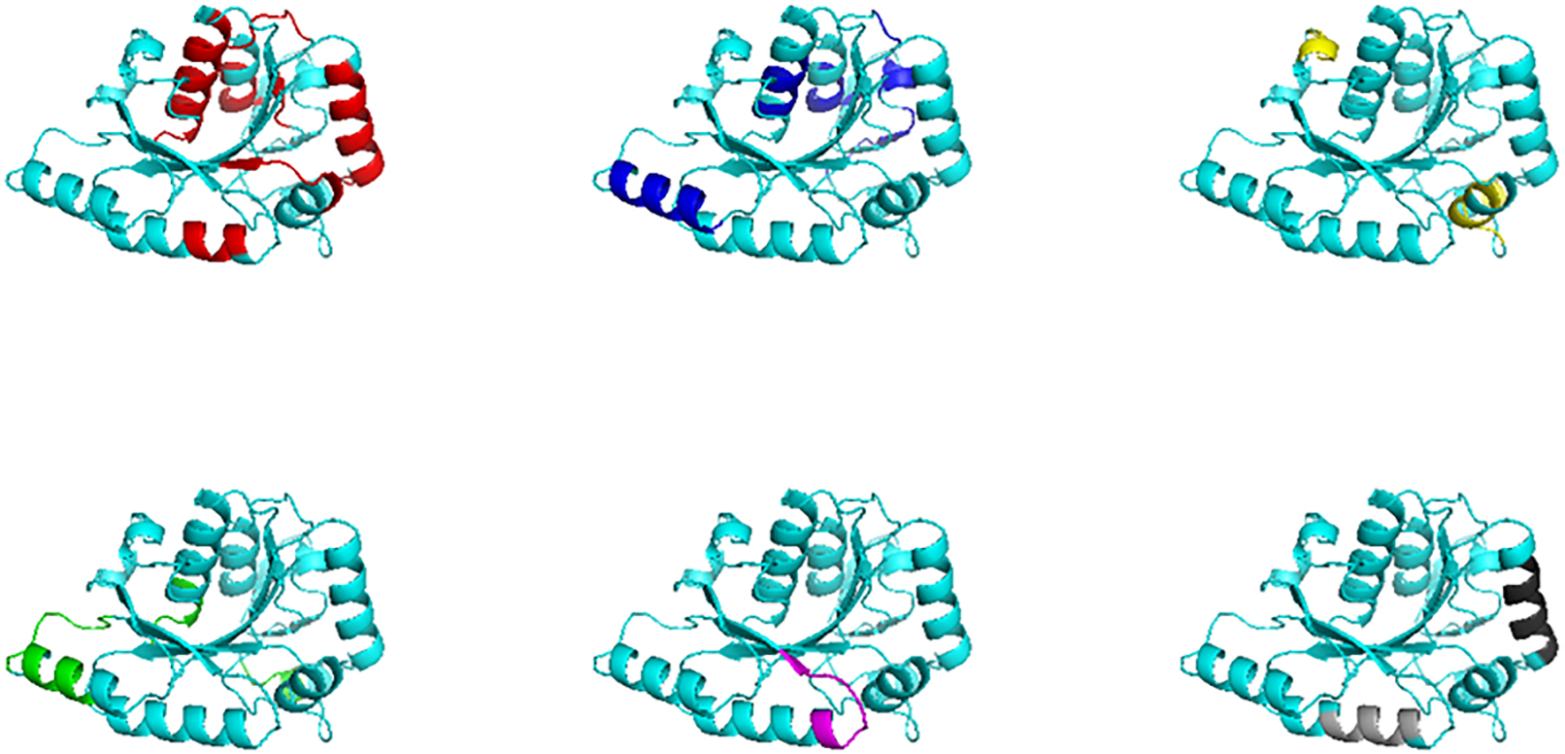
Structure of dethiobiotin synthase (PDB ID 1byi) represented in cartoon representation showing mapped BPs. Red indicates ACE inhibitor BP activity (14), blue antibacterial (4), yellow inmunomodulating (3), green antioxidative (3), magenta neuropeptide (1), light grey chemotactic (1) and dark grey stimulating activities (1) (as derived from BIOPEP database). It is possible to see that different activities have similar location. BPs found in different proteins of the same superfamily have been mapped on a representative structure (1byi) accordingly to CATH (see Materials and methods).

Using structural superfamilies with 5 or more biopeptides mapped in the corresponding representative fold we found that the different biopeptides, also those with different biological functions, overlap in certain regions of the structure. For example, in Fig 6, we represent 7 different BP activity classes indicated with different colours (ACE inhibitor, antibacterial, antioxidative, chemotactic, inmunomodulating, neuropeptide and stimulating) mapped onto the representative fold. It is possible to see that different BPs are mapped in similar regions of the protein fold. Similar results are found in different protein families (S1 and S2 Figs). These figures corresponds to well populated folds with several BP mapped (different colours indicate different biological activity (as indicated also in Table 2). When we explored general characters of these regions, we were unable to find any correlation between occurrence of biopeptides in the surface or buried areas of mapped proteins, neither a correlation with the type of biopeptide’s biological activity. It is then possible that BPs at the superfamily level occur in some “hot-spots” regions.

**Table 2:** Detail of the sequence and activity of biopeptides of the first four superfamilies with the highest number of biopeptides. The activity corresponds to the classification in BIOPEP.

CATH	Structures/domains	Number of BP	Sequences of each BP	Activity
3.40.50.300	215	35	GKKVLQ; DYGLYP; PQEVLP; PQEVLP; KVLILA; RADHPF; LAHKAL; KVLAGM; GLDIQK; VTSTAV; IKPLNY; FQKVVA; VPQPIP; VIEKYP; KVREGT; VHLPPP;	Ace Inhibitor
			LKKISQ	Ace Inhibitor/Antibacterial
			RRPYIL; KIPYIL	Anti Inflammatory
			HLPLPL	Antiamnestic
			STVATL; ALCSEK	Antibacterial
			IEAEGE; LLPHHH; QYDQGV; GALAAH	Antioxidative
			LGTIPG; VGVAPG	Chemotactic
			SIKVAV	Immunomodulating/Anticancer
			VGGIPY	Immunomodulating
			FFGLMG; KRQHPG	Neuropeptide
			YLGYLE; RYLGYL; GGFLTRH	Opioid
			SFLLRN	Stimulating
3.40.50.720	309	25	VTSTAV; PANIKWGD; PSKIKWGD; QSLVYP; PQEVLP; PANLPWGSSNV; VLAQYK; VIEKYP; LAHKAL; EPKAIP; KVLAGM; GLDIQK	Ace Inhibitor
			LKKISQ	Ace Inhibitor/Antibacterial
			ALCSEK	Antibacterial
			GALAAH; LGFEYY; PKAVHE; ISELGW	Antioxidative
			RGDSPA	Antithrombotic
			VGVAPG; LGTIPG; PGAIPG	Chemotactic
			LVCYPQ	Immunomodulating
			SIKVAV	Immunomodulating/Anticancer
			EVQKQLQ	Neuropeptide
3.20.20.70	35	18	KVLILA; VLPYPV; PQEVLP; GKKVLQ; LAHKAL; KVLPVP; YLYEIA; VTSTAV	Ace Inhibitor
			LKKISQ	Ace Inhibitor/Antibacterial
			STVATL	Antibacterial
			YFYPEL; GALAAH; PKAVHE; EELDNALN; YGYTGA	Antioxidative
			PGAIPG; VGVAPG	Chemotactic
			SIKVAV	Immunomodulating/Anticancer
2.60.120.10	246	17	KVLILA; NWGPLV; YLAGNQ; YQEPVL	Ace Inhibitor
			LSPFWNINA	Antiamnestic
			STVATL	Antibacterial
			AIRQGDVF; KHNRGDEF; LLPHHADADY; LVNPHDHQN; VIPAGYP; VLEANPRSF; YFPVGGDRPESF	Antioxidative
			RGDSPA	Antithrombotic
			HCQRPR	Immunomodulating
			EITPEKNPQLR; VAWWMY	Inhibitor

## Conclusions

Taking into account that all proteins may be considered to contain BP [32,33] we studied BPs distribution in sequence and protein structural space. We followed the simpler possible analysis to identify putative BPs in protein sequences using 100% identity matches to well established BPs deposited in BIOPEP database. After our large-scale BP mapping, two major results emerged from our analysis. The first one is that proteins containing high number of BPs (≥5) belong to superfamilies very diverse in their sequences and in their biological functions (Fig 4). Each superfamily is represented by a given protein fold which is conserved among all the members as an evidence of homology between all their sequences. However, in those superfamilies with proteins with 5 or more BPs in their sequences these representative fold shows a higher structural variability (Fig 5). Protein folds associated to a great number of homologous families with the same or different biological function have been called “superfolds” as derived from a landmark work by Orengo and collaborators [30,34]. As it was estimated that there is a limited number of protein folds in nature [35] it follows that some of them could be overrepresented given place to the presence of superfolds. Also, they have been associated with an differentially increased thermostability [36] and with a lesser representation in microbial superkingdoms Archea and Bacteria in reference with those of Eukarya [37]. The second main result is that in these proteins with 5 or more BPs in their sequences, BPs with different activities tend to locate in similar regions (putative hotspots) when are mapped in the representative fold of the superfamily which they belong to (Fig 6, S1 and S2 Figs).

As we mentioned above, BPs sequences are not expected to evolve under a selective pressure in order to preserve its corresponding BP biological activity. BPs express their activity when are released by proteolytic cleavage or other types of fragmentation in the digestive tract [38]. As has been previously suggested, BPs environment, flanking solvent exposition regions as well the presence of hydrophilic regions, could favour the action of proteinases for BPs release [39]. Taking into account the results obtained in our work, presence of superfolds and putative hotspots for those proteins with more than 5 BPs, suggest the presence of common mechanisms to release the BPs during the digestion. Although structurally diverse as suggested in Fig 5, superfolds, and folds in general, could conserve hydrophilic/hydrophobic regions in order to conserve the same fold [40] and then solvent exposed areas are also conserved. The observation of putative hotspots were different activity BPs co-localize, possibly results from the conservation of solvent exposed regions and putative targets of proteolytic enzymes.

Analysis of protein fold distribution containing BPs (Fig 3) could certainly reveal bias composition of sequences in databases and mostly agree with previous results [41,42]. In fact, rubisco (CATH id 3.30.70.150), the most abundant protein on earth, has been proposed as a sustainable source of bioactive peptides, supported by *in vitro* and *in vivo* analyzes [43].

One of the most common procedure to predict the presence of BPs in proteins is the use of sequence similarity searches using well curated databases [21] followed by in vitro hydrolysis and/or simulated digestion and identification [44]. Our results also indicate two features that could improve target-directed search of BPs in proteins. Identification of the fold in which a given sequence would possible fold, is nowadays routinely used in widely used homology modeling techniques [45,46]. Fold assignment techniques have also been developed to be used at the proteomic level [47]. Identification of superfamilies containing superfolds could increase the detection rate of BPs in wet-screening procedures. Additionally, these detected superfolds could also contain more than one type of BPs having different biological activities as derived from our results.

Although it is difficult to put our results in an evolutionary context, just because BPs are not independent of the evolutionary constraints of the whole protein they belong to, our results could help in the design of new bioinformatics pipelines to improve predictions of BPs occurrences.

## Supporting information

S1 Fig Structure of dethiobiotin synthase (PDB ID 1c0p, domain A01 accordingly with CATH (1c0pA01)) represented in cartoon representation showing mapped BPs. Red indicates ACE inhibitor BP activity (12), green antibacterial(1), blue antioxidative (5) and orange chemostatic activities (13) (as derived from Biopep database). It is possible to see that different activities have similar location. BPs found in different proteins of the same superfamily have been mapped on a representative structure (1c0p) accordingly to CATH.
(TIF)

S2 Fig Structure of Triosephosphate Isomerase (PDB ID 2vxn, domain A00 accordingly with CATH (2vxnA00)) represented in cartoon representation showing mapped BPs. Red indicates ACE inhibitor BP activity (6), green antibacterial (1), blue antioxidative (2) and orange chemostatic activities (1) (as derived from Biopep database). It is possible to see that different activities have similar location. BPs found in different proteins of the same superfamily have been mapped on a representative structure (12vxn) accordingly to CATH.
(TIF)

S1 Table List of complete CATH domains found in the assignation of structure of the sequences with at least one peptide. (PDF)

S2 Table Number of biopeptides and proteins associated with a given protein fold (CATH id). (PDF)
