# Supplementary figures and images for: Large-scale mapping of bioactive peptides in structural and sequence space
Source: PLoS One. 2018 Jan 19;13(1):e0191063. doi: 10.1371/journal.pone.0191063 (PMC5774755; doi:10.1371/journal.pone.0191063)

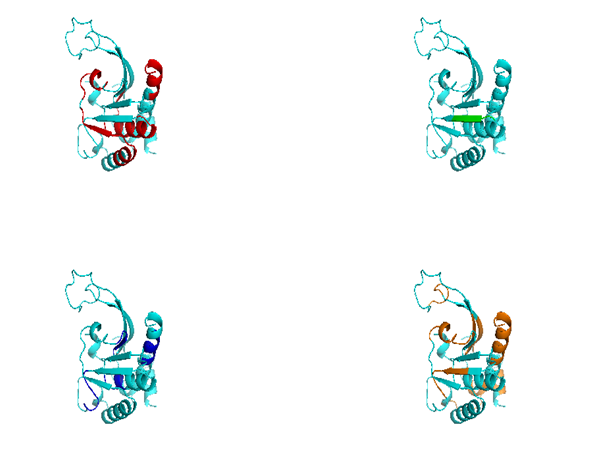

Supplement: S1 Fig — Red indicates ACE inhibitor BP activity (12), green antibacterial(1), blue antioxidative (5) and orange chemostatic activities (13) (as derived from Biopep database). It is possible to see that different activities have similar location. BPs found in different proteins of the same superfamily have been mapped on a representative structure (1c0p) accordingly to CATH. (TIF) [file pone.0191063.s001.tif]

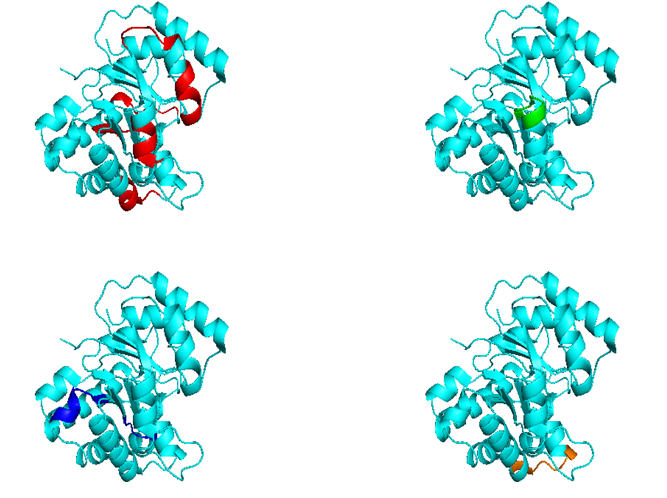

Supplement: S2 Fig — Red indicates ACE inhibitor BP activity (6), green antibacterial (1), blue antioxidative (2) and orange chemostatic activities (1) (as derived from Biopep database). It is possible to see that different activities have similar location. BPs found in different proteins of the same superfamily have been mapped on a representative structure (12vxn) accordingly to CATH. (TIF) [file pone.0191063.s002.tif]
