# Supplementary material for: Large-scale mapping of bioactive peptides in structural and sequence space
Source: PLoS One. 2018 Jan 19;13(1):e0191063. doi: 10.1371/journal.pone.0191063 (PMC5774755; doi:10.1371/journal.pone.0191063)
Supplement: S2 Table — (PDF) [file pone.0191063.s004.pdf]

**CATH**	Proteins	Percentage of proteins with assigned fold	Number of BP
3.40.50.300	215	0.370	35
3.40.50.720	309	0.531	25
3.20.20.70	35	0.060	18
2.60.120.10	246	0.423	17
3.40.50.1820	22	0.038	17
3.20.20.80	126	0.217	13
3.40.50.150	144	0.248	12
3.50.50.60	56	0.096	10
3.90.226.10	237	0.407	9
1.10.630.10	18	0.031	9
3.40.190.10	322	0.554	8
2.60.40.10	161	0.277	8
1.10.530.10	151	0.260	8
3.40.50.200	53	0.091	8
3.40.50.2300	41	0.070	8
3.40.640.10	28	0.048	8
3.40.50.620	12	0.021	8
3.30.420.40	2989	5.139	7
3.40.605.10	12	0.021	7
2.40.50.140	8	0.014	7
3.20.20.30	6	0.010	7
3.80.10.10	39	0.067	6
1.10.510.10	33	0.057	6
3.30.930.10	20	0.034	6
3.40.30.10	12	0.021	6
1.10.490.10	884	1.520	5
2.80.10.50	148	0.254	5
3.30.200.20	36	0.062	5
2.40.128.20	30	0.052	5
3.20.20.140	30	0.052	5
3.40.50.880	13	0.022	5
3.40.1190.20	10	0.017	5
3.40.630.10	10	0.017	5
3.40.50.80	8	0.014	5
3.40.710.10	7	0.012	5
3.30.420.10	5	0.009	5
1.10.565.10	39	0.067	4
2.30.39.10	32	0.055	4
1.20.1070.10	31	0.053	4
3.60.21.10	29	0.050	4
3.40.50.1970	13	0.022	4
2.40.10.10	11	0.019	4
3.40.309.10	11	0.019	4
3.30.565.10	10	0.017	4
3.40.50.970	10	0.017	4
1.10.3720.10	9	0.015	4
3.10.310.10	9	0.015	4
3.90.1300.10	9	0.015	4
1.20.1560.10	7	0.012	4
2.40.170.20	7	0.012	4
3.20.20.60	5	0.009	4
1.10.8.60	4	0.007	4
3.40.47.10	4	0.007	4
3.60.15.10	4	0.007	4
4.10.230.10	63	0.108	3
1.10.238.10	55	0.095	3
3.10.250.10	39	0.067	3
3.10.50.40	31	0.053	3
3.30.365.10	29	0.050	3
3.40.50.2020	26	0.045	3
3.40.980.10	17	0.029	3
3.90.550.10	15	0.026	3
1.10.10.10	14	0.024	3
3.10.450.10	12	0.021	3
3.40.50.1240	12	0.021	3
3.20.20.300	8	0.014	3
3.40.1190.10	8	0.014	3
3.30.450.20	7	0.012	3
3.30.470.20	7	0.012	3

3.90.1150.10	7	0.012	3
2.130.10.10	6	0.010	3
4.10.70.10	6	0.010	3
3.40.109.10	5	0.009	3
3.90.180.10	5	0.009	3
3.40.50.170	4	0.007	3
3.60.20.10	4	0.007	3
3.90.190.10	4	0.007	3
1.10.760.10	3	0.005	3
1.25.40.10	3	0.005	3
3.40.1280.10	3	0.005	3
3.40.50.1360	3	0.005	3
3.40.50.1980	3	0.005	3
2.60.120.20	1	0.002	3
3.20.20.10	1	0.002	3
2.170.40.20	12388	21.297	2
2.60.40.720	313	0.538	2
3.30.1340.10	76	0.131	2
3.50.7.10	65	0.112	2
1.10.246.10	46	0.079	2
3.30.1330.10	32	0.055	2
3.30.360.10	25	0.043	2
1.20.5.110	24	0.041	2
1.20.58.70	24	0.041	2
1.50.40.10	21	0.036	2
2.60.120.200	19	0.033	2
3.20.20.120	17	0.029	2
3.40.50.1100	16	0.028	2
3.40.810.20	14	0.024	2
3.30.70.270	13	0.022	2
3.10.105.10	11	0.019	2
3.90.870.10	11	0.019	2
3.30.450.90	10	0.017	2
1.25.10.10	9	0.015	2
3.40.850.10	9	0.015	2
3.30.497.10	8	0.014	2
1.10.3080.10	6	0.010	2
1.20.1050.10	6	0.010	2
1.10.560.10	5	0.009	2
1.10.600.10	4	0.007	2
3.20.20.100	4	0.007	2
3.30.1060.10	4	0.007	2
3.40.50.10540	4	0.007	2
3.90.245.10	4	0.007	2
2.30.110.10	3	0.005	2
3.10.129.10	3	0.005	2
3.20.20.330	3	0.005	2
3.30.300.20	3	0.005	2
3.40.1030.10	3	0.005	2
3.40.50.2000	3	0.005	2
3.40.50.980	3	0.005	2
3.40.630.20	3	0.005	2
3.60.40.10	3	0.005	2
1.10.290.10	2	0.003	2
1.10.3470.10	2	0.003	2
1.10.520.10	2	0.003	2
1.20.120.80	2	0.003	2
1.20.210.10	2	0.003	2
1.20.810.10	2	0.003	2
2.40.30.10	2	0.003	2
2.60.40.820	2	0.003	2
2.70.98.10	2	0.003	2
3.20.20.240	2	0.003	2
3.20.20.370	2	0.003	2
3.30.1360.30	2	0.003	2
3.30.300.30	2	0.003	2
2.50.20.10	1	0.002	2
3.30.70.150	19543	33.598	1
1.10.287.210	15978	27.469	1
2.30.36.70	969	1.666	1
3.40.50.410	181	0.311	1
1.20.5.340	154	0.265	1

1.20.5.350	133	0.229	1
4.10.270.10	103	0.177	1
1.10.2060.10	69	0.119	1
2.30.29.30	40	0.069	1
1.10.20.10	39	0.067	1
3.30.590.10	38	0.065	1
2.40.180.10	33	0.057	1
1.10.220.10	27	0.046	1
2.60.120.290	26	0.045	1
3.30.190.10	26	0.045	1
1.20.5.170	21	0.036	1
1.10.540.10	18	0.031	1
3.40.366.10	16	0.028	1
1.20.5.50	15	0.026	1
1.50.30.10	15	0.026	1
1.20.140.10	14	0.024	1
3.40.50.800	14	0.024	1
4.10.372.10	14	0.024	1
1.10.150.90	12	0.021	1
3.15.10.10	11	0.019	1
3.30.260.10	9	0.015	1
2.30.30.310	8	0.014	1
3.30.950.10	8	0.014	1
3.40.50.670	8	0.014	1
1.10.580.10	7	0.012	1
3.40.50.10470	7	0.012	1
1.20.1050.60	6	0.010	1
3.10.20.90	5	0.009	1
3.20.19.10	5	0.009	1
3.40.1410.10	5	0.009	1
1.10.220.20	4	0.007	1
2.30.30.190	4	0.007	1
3.10.540.10	4	0.007	1
3.30.559.10	4	0.007	1
3.30.60.10	4	0.007	1
3.40.367.20	4	0.007	1
3.40.600.10	4	0.007	1
1.10.120.10	3	0.005	1
1.10.468.10	3	0.005	1
1.50.10.100	3	0.005	1
2.160.20.10	3	0.005	1
2.170.120.12	3	0.005	1
2.40.33.10	3	0.005	1
3.10.580.10	3	0.005	1
3.40.470.10	3	0.005	1
3.40.50.1400	3	0.005	1
3.90.1510.10	3	0.005	1
3.90.190.20	3	0.005	1
1.10.1200.10	2	0.003	1
1.10.8.280	2	0.003	1
1.10.860.10	2	0.003	1
1.20.1610.10	2	0.003	1
1.20.200.10	2	0.003	1
2.140.10.10	2	0.003	1
2.140.10.30	2	0.003	1
2.30.38.10	2	0.003	1
2.40.160.60	2	0.003	1
2.60.300.12	2	0.003	1
3.20.20.210	2	0.003	1
3.30.1130.10	2	0.003	1
3.30.230.40	2	0.003	1
3.30.428.10	2	0.003	1
3.30.450.80	2	0.003	1
3.30.70.330	2	0.003	1
3.30.70.930	2	0.003	1
3.40.718.10	2	0.003	1
3.40.720.10	2	0.003	1
3.60.110.10	2	0.003	1
3.90.110.10	2	0.003	1
3.90.700.10	2	0.003	1
1.10.10.670	1	0.002	1
1.10.1000.11	1	0.002	1

1.10.1030.10	1	0.002	1
1.10.1280.10	1	0.002	1
1.10.150.20	1	0.002	1
1.10.1500.10	1	0.002	1
1.10.1670.10	1	0.002	1
1.10.1750.10	1	0.002	1
1.10.1780.10	1	0.002	1
1.10.1900.10	1	0.002	1
1.10.1900.20	1	0.002	1
1.10.287.110	1	0.002	1
1.10.3150.10	1	0.002	1
1.10.3370.10	1	0.002	1
1.10.340.30	1	0.002	1
1.10.357.10	1	0.002	1
1.10.3860.10	1	0.002	1
1.10.400.20	1	0.002	1
1.10.405.10	1	0.002	1
1.10.420.10	1	0.002	1
1.10.601.10	1	0.002	1
1.20.120.330	1	0.002	1
1.20.1260.10	1	0.002	1
1.20.1340.10	1	0.002	1
1.20.1370.10	1	0.002	1
1.20.1600.10	1	0.002	1
1.20.990.10	1	0.002	1
1.25.40.20	1	0.002	1
1.25.40.90	1	0.002	1
2.10.110.10	1	0.002	1
2.140.10.20	1	0.002	1
2.160.10.10	1	0.002	1
2.30.30.110	1	0.002	1
2.40.10.170	1	0.002	1
2.40.128.130	1	0.002	1
2.40.160.50	1	0.002	1
2.40.270.10	1	0.002	1
2.40.40.20	1	0.002	1
2.60.120.260	1	0.002	1
2.60.120.590	1	0.002	1
2.60.15.10	1	0.002	1
2.60.20.30	1	0.002	1
2.60.200.30	1	0.002	1
2.60.260.20	1	0.002	1
2.60.40.1360	1	0.002	1
2.60.40.200	1	0.002	1
2.60.40.320	1	0.002	1
2.60.40.340	1	0.002	1
2.70.10.10	1	0.002	1
2.70.210.12	1	0.002	1
3.10.110.10	1	0.002	1
3.10.20.30	1	0.002	1
3.10.200.10	1	0.002	1
3.10.400.10	1	0.002	1
3.10.450.40	1	0.002	1
3.20.20.150	1	0.002	1
3.20.20.190	1	0.002	1
3.20.20.20	1	0.002	1
3.20.20.410	1	0.002	1
3.20.20.450	1	0.002	1
3.30.1120.10	1	0.002	1
3.30.1330.40	1	0.002	1
3.30.1370.60	1	0.002	1
3.30.1490.10	1	0.002	1
3.30.2080.10	1	0.002	1
3.30.230.10	1	0.002	1
3.30.230.60	1	0.002	1
3.30.230.70	1	0.002	1
3.30.379.10	1	0.002	1
3.30.420.100	1	0.002	1
3.30.420.140	1	0.002	1
3.30.429.10	1	0.002	1
3.30.450.30	1	0.002	1
3.30.470.10	1	0.002	1

3.30.479.20	1	0.002	1
3.30.530.20	1	0.002	1
3.30.70.1230	1	0.002	1
3.30.70.370	1	0.002	1
3.30.70.580	1	0.002	1
3.30.70.600	1	0.002	1
3.30.70.890	1	0.002	1
3.30.830.10	1	0.002	1
3.30.870.10	1	0.002	1
3.30.9.10	1	0.002	1
3.30.990.10	1	0.002	1
3.40.1110.10	1	0.002	1
3.40.192.10	1	0.002	1
3.40.225.10	1	0.002	1
3.40.33.10	1	0.002	1
3.40.50.1000	1	0.002	1
3.40.50.10190	1	0.002	1
3.40.50.10260	1	0.002	1
3.40.50.10320	1	0.002	1
3.40.50.10490	1	0.002	1
3.40.50.1470	1	0.002	1
3.40.50.1580	1	0.002	1
3.40.50.180	1	0.002	1
3.40.50.1860	1	0.002	1
3.40.50.2030	1	0.002	1
3.40.50.360	1	0.002	1
3.40.50.790	1	0.002	1
3.40.50.960	1	0.002	1
3.40.630.30	1	0.002	1
3.40.800.10	1	0.002	1
3.40.950.10	1	0.002	1
3.50.30.10	1	0.002	1
3.50.30.50	1	0.002	1
3.60.120.10	1	0.002	1
3.60.130.10	1	0.002	1
3.60.70.12	1	0.002	1
3.90.215.10	1	0.002	1
3.90.230.10	1	0.002	1
3.90.25.10	1	0.002	1
3.90.260.10	1	0.002	1
3.90.420.10	1	0.002	1
3.90.45.10	1	0.002	1
3.90.740.10	1	0.002	1
3.90.800.10	1	0.002	1
3.90.920.10	1	0.002	1
3.90.950.10	1	0.002	1
3.90.960.10	1	0.002	1
4.10.410.10	1	0.002	1
